# Supplementary material for: A single N1-methyladenosine on the large ribosomal subunit rRNA impacts locally its structure and the translation of key metabolic enzymes
Source: Sci Rep. 2018 Aug 9;8:11904. doi: 10.1038/s41598-018-30383-z (PMC6085284; doi:10.1038/s41598-018-30383-z)
Supplement: Supplementary file 1 — Supplementary Data [file 41598_2018_30383_MOESM1_ESM.docx]

**A single N^1^-methyladenosine on the large ribosomal subunit rRNA impacts locally its structure and the translation of key metabolic enzymes**

Sunny Sharma^1,2,3, §, #^, Johannes David Hartmann^1, §^, Peter Watzinger^1^, Arvid Klepper^1^, Christian Peifer^1^, Peter Kötter^1^, Denis LJ Lafontaine^2^, Karl-Dieter Entian^1, #^

^1^ Institute of Molecular Biosciences, Goethe University, Frankfurt am Main, D-60438, Germany

^2^ RNA Molecular Biology, Fonds National de la Recherche (F.R.S./FNRS), ULB-Cancer Research Center (U-CRC), Center for Microscopy and Molecular Imaging (CMMI), Université Libre de Bruxelles (ULB), BioPark campus, B-6041 Gosselies-Charleroi, Belgium

^3^ Present address – Massachusetts General Hospital Centre for Cancer Research and Department of Medicine, Harvard Medical School, Boston -02129, USA

^§^ Joint first author

^#^ Corresponding author: Correspondence should be addressed to Dr. Sunny Sharma: [ssharma33@mgh.harvard.edu](mailto:ssharma33@mgh.harvard.edu) and Prof. Dr. Karl-Dieter Entian: [entian@bio.uni-frankfurt.de](mailto:entian@bio.uni-frankfurt.de) and

**Supplementary Figures**


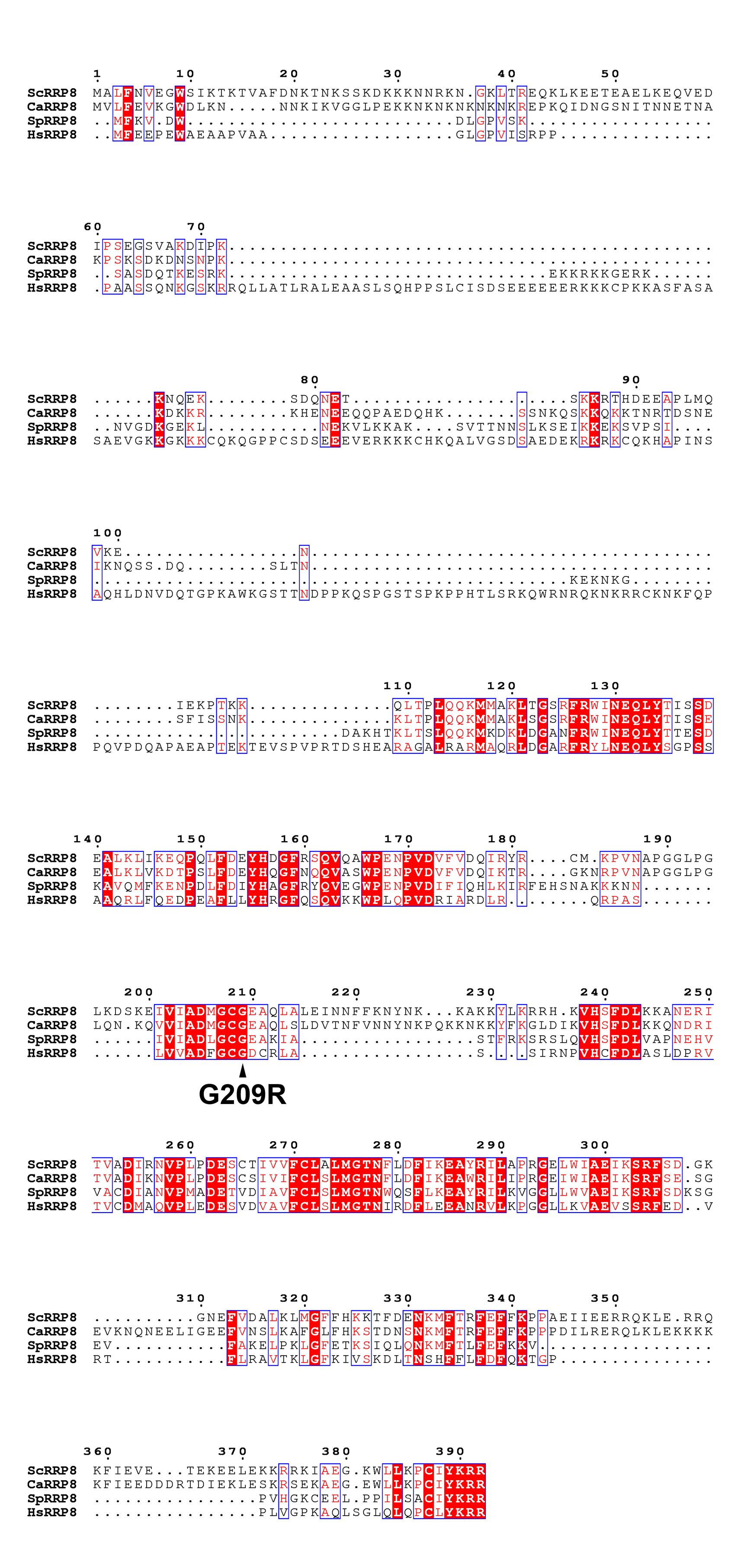


Figure S1 **Rrp8 is a highly-conserved protein among eukaryotes.** Amino acid sequence alignment of Rrp8 from S. cerevisiae (Sc), C. albicans (ca), S. pombe (Sp) and Homo sapiens (Hs). All multi-alignments were performed with the open-source software T-coffee (32). EsPript was used to visualize alignments.


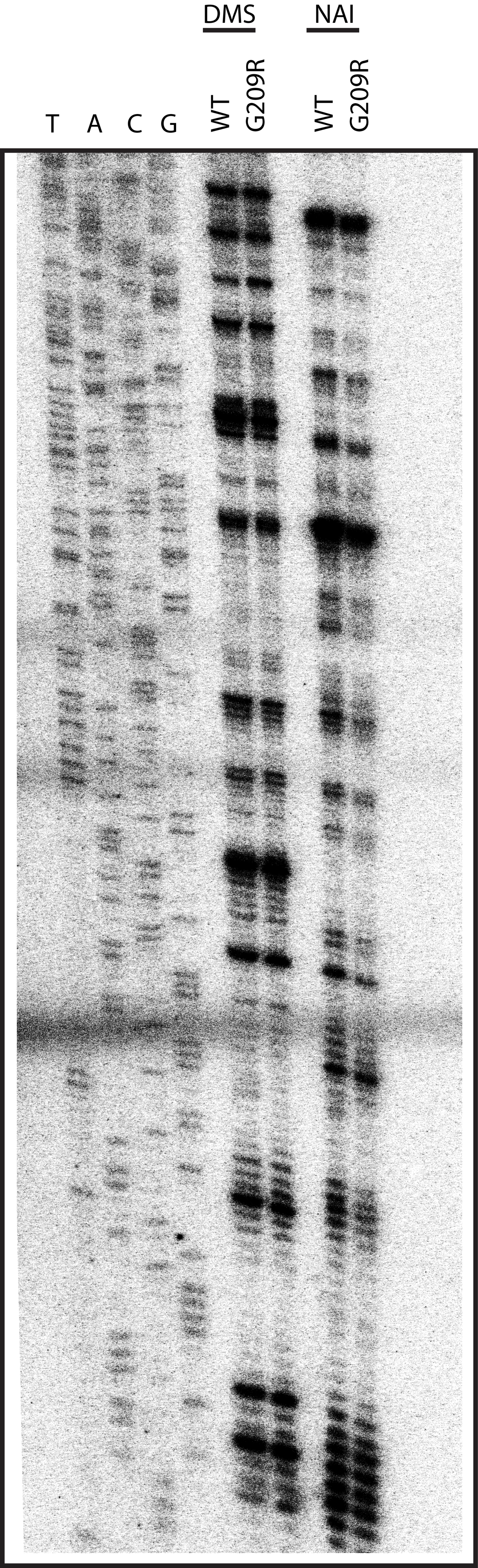


Figure S2 **Loss of m^1^A_645_ does not influence the conformation of helix 72**. Representative gel showing structure probing with DMS or SHAPE analysis with NAI in the rrp8^G209R^ loss of methylation mutant. ^32^P-labeled primer (helix72_StrPrb) complementary to nucleotides 2428 to 2448 of human 28S rRNA were used for the analysis.


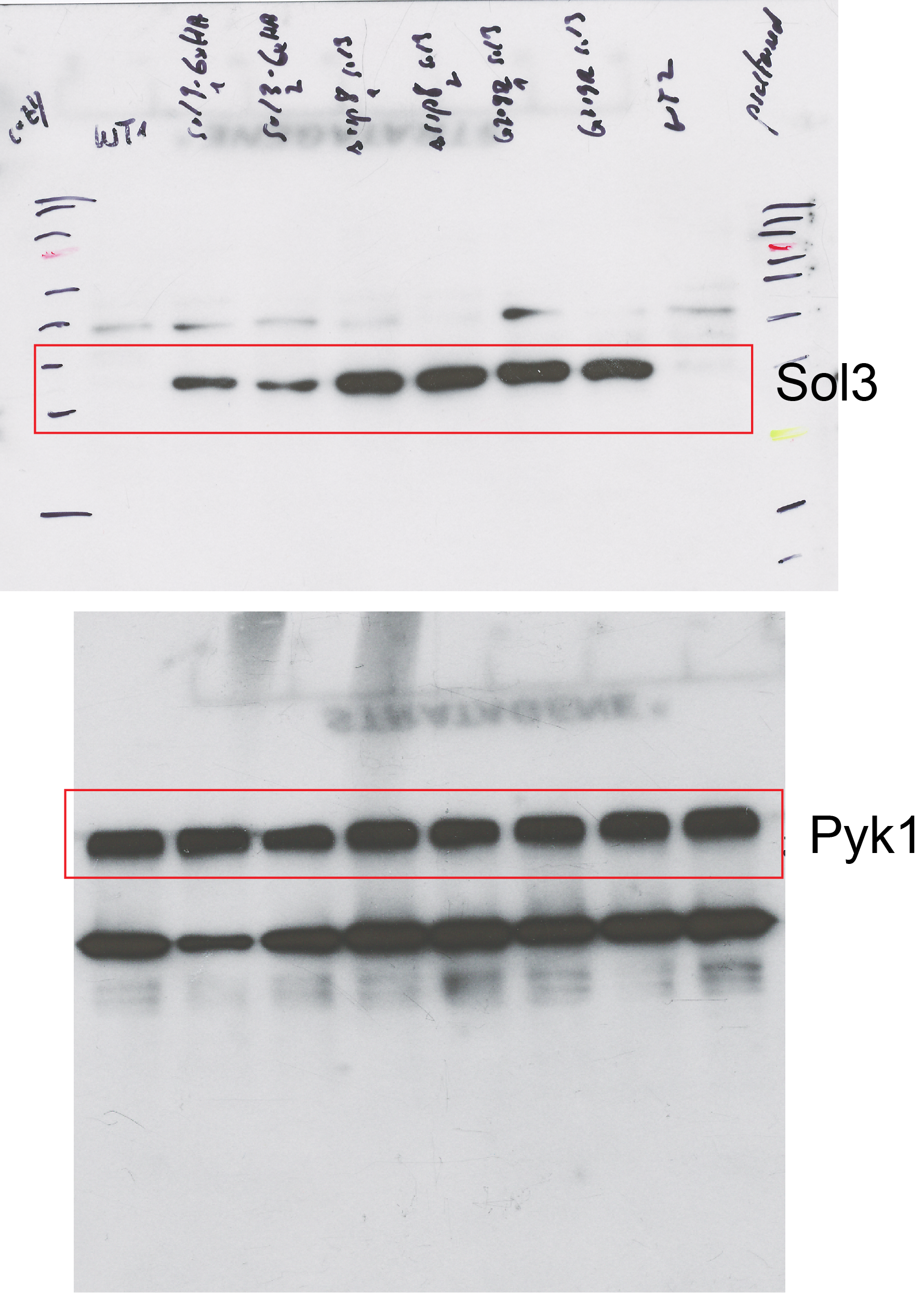


Figure S3. **Uncropped Western blots, shown in Fig 6C**

**Supplementary Tables**

**Table S1** *S. cerevisiae* strains used within this work

| Strain | Genotype | Reference |
| --- | --- | --- |
| CEN.PK2-1C | MATa; ura3-52; his3-Δ1; leu2-3,112; trp1-289; MAL2-8^c^; SUC2 | Entian, K.-D. & Kötter, P. Yeast Genetic Strain and Plasmid Collections. *Methods in Microbiology Academic Press Ltd.* **36,** 629–666 (2007). |
| CEN.PK1265-4D | MATa; ura3-52; his3-Δ1; leu2-3,112; trp1-289; MAL2-8c; SUC2;  rrp8(51,1050)::loxP-natMX4-loxP | This study |
| CEN.CP6-2A | MATa; ura3-52; his3-Δ1; leu2-3,112; trp1-289; MAL2-8c; SUC2;  rrp8(G682C) | Peifer, C. *et al.* Yeast Rrp8p, a novel methyltransferase responsible for m1A 645 base modification of 25S rRNA. *Nucleic acids research* **41,** 1151–1163 (2013). |
| CEN.PK1174-16A | MATa; ura3-52; his3-Δ1; leu2-3,112; trp1-289; MAL2-8^c^; SUC2;  sol3(STOP,-40)::6xHA-kanMX4 | This study |
| CEN.MB678-2A | MATa; ura3-52; his3-Δ1; leu2-3,112; trp1-289; MAL2-8c; SUC2;  rrp8(788-895)::loxP-SpHIS5-loxP | Peifer, C. *et al.* Yeast Rrp8p, a novel methyltransferase responsible for m1A 645 base modification of 25S rRNA. *Nucleic acids research* **41,** 1151–1163 (2013). |
| CEN.PK1175-1C | MATa; ura3-52; his3-Δ1; leu2-3,112; trp1-289; MAL2-8c; SUC2;  rrp8(788,895)::loxP-kanMX4-loxP  sol3(STOP,-40)::6xHA-kanMX4 | This study (from crossing CEN.MB677-1C x CEN.PK1174-2B) |
| CEN.DH37-4C | MATa; ura3-52; his3-Δ1; leu2-3,  112; trp1-289; MAL2-8c; SUC2;  sol3(STOP,-40)::6xHA-loxP-KanMX-loxP rrp8(G682C) | This study (from crossing (CEN.CP6-2C und CEN.PK1174-2D) |
| Y00000 | BY4741; MATa; his3Δ1; leu2Δ0; lys2Δ0; ura3Δ0 | EUROSCARF |
| Y04018 | BY4741; MATa; ura3Δ0; leu2Δ0; his3Δ1; met15Δ0; YDR083w::kanMX4 | EUROSCARF |
| Y02857 | BY4741; MATa; ura3Δ0; leu2Δ0; his3Δ1; met15Δ0; YHR163w::kanMX4 | EUROSCARF |
| BY.PK1177-1C | MATa; ura3Δ0; leu2Δ0; his3Δ1; met15Δ0; YHR163w::kanMX4 YDR083w::kanMX4 | This study (from crossing Y14018 x Y02857) |
| Y01971 | BY4741; MATa; ura3Δ0; leu2Δ0; his3Δ1; met15Δ0; YNL241c::kanMX4 | EUROSCARF |
| BY.PK1184-9B | MATa; ura3Δ0; leu2Δ0; his3Δ1; met15Δ0; YNL241c::kanMX4 YDR083w::kanMX4 | This study (from crossing Y14018 x Y01971) |
| Y06998 | BY4741; MATa; ura3Δ0; leu2Δ0; his3Δ1; met15Δ0; YGR248w::kanMX4 | EUROSCARF |
| BY.PK1186-12A | MATa; ura3Δ0; leu2Δ0; his3Δ1; met15Δ0; YGR248w::kanMX4 YDR083w::kanMX4 | This study (from crossing Y14018 x Y06998) |
| BY.DH4-1C | MATa; ura3Δ0; leu2Δ0; his3Δ1; met15Δ0; YDR083w::natMX4 | This study |
| BY.PK1179-5D | MATa; ura3Δ0; leu2Δ0; his3Δ1; met15Δ0;YHR163w::kanMX4 YGR248w::kanMX4 | This study (from crossing Y02857 x Y16998) |
| BY.DH5-3B | MATa; ura3Δ0; leu2Δ0; his3Δ1; met15Δ0;YDR083w::natMX4 YHR163w::kanMX4 YGR248w::kanMX4 | This study (from crossing BY.PK1179-5D X BY.DH4-1C) |

**Table S2** Plasmids used within this work

| Plasmids | Reference |
| --- | --- |
| pPK468 | Peter Kötter, unpublished data |
| pPK468-CaRRP8 | this study |
| pPK468-SpRRP8 | this study |
| pPK468-NML | this study |
| pPK468-nml^G318R^ | this study |

**Table S3** Oligonucleotides used within this work

| name | sequence | application |
| --- | --- | --- |
| RRP8-S1 | TTAACGTAGAAGGTTGGTCTATTAAGACAAAAACCGTCGCCAGCTGAAGCTTCGTACGC | Deletion of *ScRRP8*-ORF |
| RRP8-S2 | CAACTTCAATAAACTTTTGTCTTCTTTCCAATTTCTGCCTGCATAGGCCACTAGTGGATCTG | Deletion of *ScRRP8*-ORF |
| CaRRP8_EcoRI | GGTGAATTCATGGTGTTATTTGAAGTTAAAGG | Construction of pPK468-CaRRP8 |
| CaRRP8_XhoI | CTACTCGAGTTACCTCCTTTTATATATACAAGG | Construction of pPK468-CaRRP8 |
| SpRRP8_Gap_for | ACCAAGAACTTAGTTTCGAATAAACACACATAAACAAACGATGTTTAAAGTAGATTGGGAC | Construction of pPK468-SpRRP8 |
| SpRRP8_Gap_rev | TATAAAAAGAAAATTTATTTAAATGCAAGATTTAAAGTAGTTATCTTCTTTTGTAAATGC | Construction of pPK468-SpRRP8 |
| HsRRP8_Gap_for | ACCAAGAACTTAGTTTCGAATAAACACACATAAACAAACGATGTTCGAAGAGCCTGAGTGG | Construction of pPK468-NML |
| HsRRP8_Gap_rev | TATAAAAAGAAAATTTATTTAAATGCAAGATTTAAAGTAGTCACCTGCGCTTGTAGAGAC | Construction of pPK468-NML |
| NML-G318R-1 | CTAGTGGTGGCTGACTTCGGCTGTCGGGATTGCCGCTTGGCTTCAAGTATC | Site-directed mutagenesis NML |
| NML-G318R-2 | GATACTTGAAGCCAAGCGGCAATCCCGACAGCCGAAGTCAGCCACCACTAG | Site-directed mutagenesis NML |
| SOL3-6HA-1 | TAATGAGGAAGCTTTTGGAAAAGTTCAAACGAAAACTTTTCGTACGCTGCAGGTCGAC | C-terminal tagging of *ScSOL3* |
| SOL3-6HA-2 | CTATGTCATTTTATAAAGTTTGGTATATATAACACGTAATATCGATGAATTCGAGCTCG | C-terminal tagging of *ScSOL3* |
| Oligo645 | CACTCGCATAGACGTTAGACTCCTTGGTCCGTGTTTCAAGACGGGCGG | Mungbean Oligo |
| ITS1(A2-A3) | GATTGCTGCAATGCCCAAAG | Northern hybridization |
| ITS2 (C1-C2) | TTGTTCGCCTAGACGCTCTC | Northern hybridization |
| ITS2 (E-C2) | CACTCACTACCAAACAGAAT | Northern hybridization |
| helix25.1_StrPrb | TCACTTTCATTACGCGTATG | Primer extension |
| Str_Prb_h25b | TCCGTGTTTCAAGACGGGCG | Primer extension |
| helix72_StrPrb | CTATGTCTCTTCACAATGTC | Primer extension |
| m^1^A_1322_ | ACGGCGGCTTTCGTGCGAGC | Primer extension |
| 166 | AGAGGAGGCAAAUAGAGUAtt | siRNA for NML depletion |
| 167 | GAAGGGUAGUACUACAAAUtt | siRNA for NML depletion |
| 168 | CGGCAAAAGAAUAAGAGAAtt | siRNA for NML depletion |
| ACT1-RT-fwd | TGAAGTGTGATGTCGATGTCCG | RT-qPCR oligo |
| ACT1-RT-rev | CGATAGATGGACCACTTTCGTCG | RT-qPCR oligo |
| ASC1-RT-fwd | GATCTCCTGGAAGTTGACTGGTG | RT-qPCR oligo |
| ASC1-RT-rev | CCAGAGATAATCATGGAAGCCTTC | RT-qPCR oligo |
| RPS5-RT-fwd | GCTGGTCGTTACGCCAACAAG | RT-qPCR oligo |
| RPS5-RT-rev | GACAGCTTGACGTCTAGCAGC | RT-qPCR oligo |
| RPS10A-RT-fwd | GGGTTACGTCAAGACTCA | RT-qPCR oligo |
| RPS10A-RT-rev | GGTACCTGGAACAATGTG | RT-qPCR oligo |
| ENO2-RT-fwd | TGGGTGCTAACGCTATCTTG | RT-qPCR oligo |
| ENO2-RT-rev | AGCACCGTATCTCTTCTTGG | RT-qPCR oligo |
| PGK1-RT-fwd | GCTCACAGAGCTCACTCTTC | RT-qPCR oligo |
| PGK1-RT-rev | CCTTGGCACCACCTAAGATG | RT-qPCR oligo |
| SOL3-RT-fwd | GAGGGCTAGTTTGACCCATC | RT-qPCR oligo |
| SOL3-RT-rev | GTCCGTCAGTGGCACAATTC | RT-qPCR oligo |
